# Molecular Characterization of Autochthonous Chikungunya Cluster in Latium Region, Italy

**DOI:** 10.3201/eid2401.171605

**Published:** 2018-01

**Authors:** Licia Bordi, Fabrizio Carletti, Eleonora Lalle, Francesca Colavita, Silvia Meschi, Antonino Di Caro, Emanuele Nicastri, Paola Scognamiglio, Francesco Vairo, Domenico Di Lallo, Vincenzo Panella, Maria R. Capobianchi, Giuseppe Ippolito, Concetta Castilletti

**Affiliations:** Lazzaro Spallanzani National Institute for Infectious Diseases, Rome, Italy (L. Bordi, F. Carletti, E. Lalle, F. Colavita, S. Meschi, A. Di Caro, E. Nicastri, P. Scognamiglio, F. Vairo, M.R. Capobianchi, G. Ippolito, C. Castilletti);; Regional Service for Surveillance and Control of Infectious Diseases, Rome (P. Scognamiglio, F. Vairo);; Regional Health and Social Policy Department, Lazio Region, Rome (D. Di Lallo, V. Panella)

**Keywords:** CHIKV, chikungunya virus, molecular characterization, Latium region, A226V, viruses, Italy

## Abstract

We report partial molecular characterization of isolates from an autochthonous chikungunya virus cluster in the Latium Region of Italy. E1 sequences from 3 patients differ substantially from sequences from the 2007 outbreak in Italy and lack the A226V substitution associated with increased viral fitness in the *Aedes* *albopictus* mosquito vector.

Local transmission of chikungunya virus (CHIKV) has been confirmed in the Lazio region of Italy, with 2 related autochthonous clusters in the cities of Anzio and Rome (*1*). This event is the second known autochthonous outbreak of CHIKV in Italy; the previous one occurred in 2007 in the Emilia Romagna region and included >205 cases of CHIKV infection during July 4–Sept 27, 2007 (*2*). During the time between the 2 epidemics in Italy, autochthonous transmissions were described in France in 2010, 2014, and 2017; these events have focused attention on this infection because of the *Aedes* *albopictus* mosquito vector establishing itself in parts of the Mediterranean basin and beyond. *Ae. albopictus* mosquitoes are assumed to be the vector in the ongoing outbreak in Italy because *Ae. aegypti* mosquitoes are not circulating in this country. The Laboratory of Virology of Lazzaro Spallanzani National Institute for Infectious Disease (INMI) in Rome, as the regional reference laboratory for arboviral infections, is in charge of CHIKV diagnosis and surveillance for the Latium region. 

CHIKV diagnosis is based on the detection of the viral genome by real-time reverse transcription PCR (RT-PCR) and of virus-specific antibodies by serologic tests. We conducted sequence analysis of endpoint PCR amplicons from selected case-patients to confirm the identity of the virus detected by real-time RT-PCR and performed virus isolation whenever possible. The first case-patient detected at INMI, a resident of Anzio (60 km from Rome) with no recent travel history abroad, was admitted to the Tropical Infectious Disease Unit on August 30, 2017, with suspected measles. Hospital staff suspected an arboviral disease on September 3, and on September 5, a serologic test was positive for chikungunya virus (E. Nicastri, unpub. data). The official notice of autochthonous cases in this region was disclosed on September 8, 2017. As of September 26, a total of 183 CHIKV infections have been reported (109 confirmed and 74 probable) (*3*), consistent with an extensive autochthonous outbreak. An intensive diagnostic campaign and backtracing of cases is under way to establish the extent of the outbreak.

Phylogenetic analyses of CHIKV strains obtained during outbreaks in different geographic regions (Réunion, Seychelles, Mauritius, Madagascar, and Mayotte) have identified the independent acquisition of a common mutation, A226V, in the E1 glycoprotein (*4*). This mutation, together with mutations M269V and D284E in E1 glycoproteins, has been described as a molecular signatures of the Indian Ocean CHIKV outbreak (*5**,**6*) The A226V mutation in particular appeared in >90% of isolates after December 2005 and has been demonstrated to increase viral fitness in *Ae. albopictus* mosquitoes, expanding the potential for CHIKV to diffuse (*5**,**6**)*). In a previous study, we described the presence of this mutation in CHIKV in specimens from patients referred to our diagnostic facility, 5 imported to Italy and 2 linked to the 2007 outbreak in Italy (*7*). In this study, we extend the genetic analysis to samples from the ongoing outbreak.

We obtained a partial sequence of the E1 coding region directly from clinical samples of 3 patients being managed at INMI. These include the patient from Anzio and 2 additional cases belonging to a family cluster of 3 cases. On September 1, a 3-year-old child, born in Rome with neither travel history nor connection with Anzio, was brought to INMI with complaints of high-grade fever, arthralgia, rash, fatigue, headache, and retro-orbital pain. His parents reported a similar febrile syndrome 24 and 48 hours later. Samples collected on September 6 for all 3 patients tested positive for CHIKV by serology and real-time RT-PCR. We obtained E1 sequences from both parents and submitted sequences for all 3 patients to GenBank (accession nos. MF988056–8). 

Further, we conducted a phylogenetic analysis that included 42 available CHIKV sequences from different parts of the world, including sequences previously analyzed in our laboratory (Technical Appendix). The analysis, based on a partial E1 sequence, showed that the virus involved in the online Latium region outbreak belongs to the broad group comprising isolates from the East/Central/South African (ECSA) clade and clusters with the Indian Ocean lineage. This finding is similar to what was observed for the 2007 Italy outbreak, but the current sequences are placed in a separate branch of the phylogenetic tree (bootstrap value 0.83). This branch also includes recent (2016) isolates from Pakistan and India, suggesting a more recent origin of the new epidemic strain, compared with the previous one affecting Italy. It is noteworthy that, unlike the isolates obtained from the 2007 outbreak in the Emilia Romagna region, the E1 sequences from the ongoing outbreak lack the A226V mutation, as do all the recent isolates placed on the same branch of the phylogenetic tree. Further study will establish the relevance of this and other genetic signatures to the fitness of the virus for the local mosquito vectors and will determine the extent of transmission cycles in humans.

**Technical Appendix.** Phylogenetic tree of E1 sequences of chikungunya virus obtained from 3 patients belonging to an ongoing cluster of autochthonous cases occurring in the Latium region of Italy, compared with reference sequences.
