# Supplementary material for: Molecular Characterization of Autochthonous Chikungunya Cluster in Latium Region, Italy
Source: Emerg Infect Dis. 2018 Jan;24(1):178–80. doi: 10.3201/eid2401.171605 (PMC5749466; doi:10.3201/eid2401.171605)
Supplement: Supplementary file 1 — Technical Appendix. Phylogenetic tree of E1 sequences of chikungunya virus obtained from 3 patients belonging to an ongoing cluster of autochthonous cases occurring in the Latium region of Italy, compared with reference sequences. [file 17-1605-Techapp-s1.pdf]

Article DOI: https://doi.org/10.3201/eid2401.171605

Molecular Characterization of Autochthonous Chikungunya Cluster in Latium Region, Italy

Technical Appendix

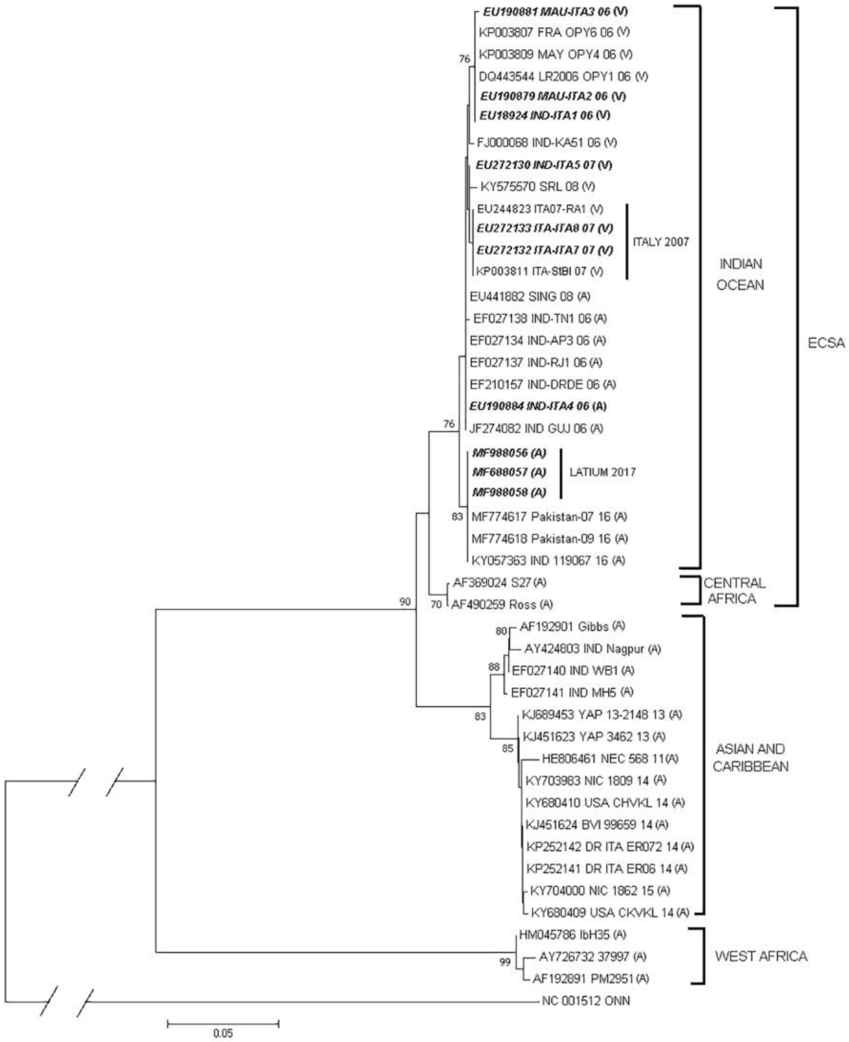

Technical Appendix Figure. Phylogenetic tree of E1 sequences of chikungunya virus obtained from 3 patients belonging to an ongoing cluster of autochthonous cases occurring in the Latium region of Italy, compared with reference sequences. The phylogenetic tree was built with the partial sequences of E1 coding region (722 bp, nucleotide positions 10178–10899 with respect to the S27 reference sequence, GenBank accession no. AF369024) obtained from these 3 patients (boldface, labeled Latium 2017;

GenBank accession nos. MF988056–8), in the context of E1 sequences representing the 3 major described CHIKV lineages: ECSA (including the Indian Ocean lineage), Asia-Caribbean, and West Africa (indicated at right). An additional 7 partial E1 human sequences previously obtained in our laboratory are noted (boldface); Italy 2007 label indicates the cluster of E1 sequences correlated with the 2007 outbreak in Italy, including 1 insect isolate (EU244823ITA07-RA1). Reference sequences are shown with GenBank accession numbers, geographic origin, and year of sampling. Letters in parentheses (A or V) indicate the amino acid observed at position 226 of E1 protein. We computed evolutionary distances using the Kimura 2-parameter method and inferred evolutionary history using the neighbor-joining method. We used the closest relative alphavirus O’nyong’nyong virus as an outgroup. Bootstraps were generated using 1,000 replicates; only those $\geq$70 are shown. Scale bars represent the genetic distance (substitution per nucleotide position). ECSA, East/Central/South African.
